# Initiation of acute pancreatitis in mice is independent of fusion between lysosomes and zymogen granules

**DOI:** 10.1007/s00018-024-05247-7

**Published:** 2024-05-06

**Authors:** Lukas Zierke, Daniel John, Marcel Gischke, Quang Trung Tran, Matthias Sendler, Frank Ulrich Weiss, Uwe T. Bornscheuer, Christoph Ritter, Markus M. Lerch, Ali A. Aghdassi

**Affiliations:** 1 Department of Medicine A, University Medicine Greifswald, Ferdinand-Sauerbruch Str, 17475 Greifswald, Germany; 2 Department of Internal Medicine, Hue University, Hue, Vietnam; 3 Institute of Biochemistry, Department of Biotechnology & Enzyme Catalysis, University of Greifswald, Greifswald, Germany; 4 Department of Pharmacy, University of Greifswald, Greifswald, Germany; 5 Ludwig-Maximilians University Munich, Munich, Germany

**Keywords:** Acute pancreatitis, Cathepsin B, Trypsinogen, Lysosome, Secretory vesicle, Co-localization

## Abstract

The co-localization of the lysosomal protease cathepsin B (CTSB) and the digestive zymogen trypsinogen is a prerequisite for the initiation of acute pancreatitis. However, the exact molecular mechanisms of co-localization are not fully understood. In this study, we investigated the role of lysosomes in the onset of acute pancreatitis by using two different experimental approaches. Using an acinar cell-specific genetic deletion of the ras-related protein Rab7, important for intracellular vesicle trafficking and fusion, we analyzed the subcellular distribution of lysosomal enzymes and the severity of pancreatitis in vivo and ex vivo. Lysosomal permeabilization was performed by the lysosomotropic agent Glycyl-L-phenylalanine 2-naphthylamide (GPN). Acinar cell-specific deletion of Rab7 increased endogenous CTSB activity and despite the lack of re-distribution of CTSB from lysosomes to the secretory vesicles, the activation of CTSB localized in the zymogen compartment still took place leading to trypsinogen activation and pancreatic injury. Disease severity was comparable to controls during the early phase but more severe at later time points. Similarly, GPN did not prevent CTSB activation inside the secretory compartment upon caerulein stimulation, while lysosomal CTSB shifted to the cytosol. Intracellular trypsinogen activation was maintained leading to acute pancreatitis similar to controls. Our results indicate that initiation of acute pancreatitis seems to be independent of the presence of lysosomes and that fusion of lysosomes and zymogen granules is dispensable for the disease onset. Intact lysosomes rather appear to have protective effects at later disease stages.

**Supplementary Information:**

The online version contains supplementary material available at 10.1007/s00018-024-05247-7.

## Introduction

Acute pancreatitis is a common gastrointestinal disease with rising incidence leading to hospitalization [1] but its underlying pathophysiology is still poorly understood. It has long been believed to begin in acinar cells where digestive proteases, in particular trypsinogen, undergo premature activation and finally cause organ injury. While under physiological conditions this activation occurs inside the duodenum and is mediated by the brush border enzyme enterokinase, intracellular trypsinogen activation is initiated by the lysosomal hydrolase cathepsin B in acute pancreatitis [2]. Newer findings challenge the CTSB-centered hypothesis for trypsinogen activation as the only determining factor for disease severity [3, 4]. Moreover, disease outcome is also related to trypsinogen autoactivation [5], ER stress due to protein misfolding [6], and cell death promoting effects of CTSB [7] as shown in acute and hereditary pancreatitis underlining the complexity of the pathogenesis of this disease.

However, this study will focus on how trypsinogen and cathepsin B, which primarily localize in secretory vesicles or lysosomes, co-localize, which is still a matter of debate [8]. Several hypotheses exist to address this question [9]. A potential mechanism of how trypsinogen and cathepsin B co-localize is the intracellular fusion of the respective subcellular compartments. The ras-related protein Rab7 is a key regulator for endo-lysosomal traffic and therefore crucial for the fusion of several intracellular vesicles [10–13]. It is expressed on the surface of both, lysosomes and zymogen granules [14]. In this study, we investigated the role of lysosomes for the initiation of acute pancreatitis. First, intracellular lysosomal transport in acinar cells was interrupted using a conditional knockout of *Rab7* in pancreatic acinar cells. Furthermore, lysosomal integrity was disrupted by permeabilization of the lysosomal membrane using the lysosomotropic chemicals Gly-Phe-β-napthylamide (GPN) [15, 16] and l-Leucyl-l-Leucyl methyl ester (LLOMe) [17], which accumulate inside the lysosomes and are hydrolyzed by cathepsin C (CTSC). The cleavage products retain within the lysosome and cause membrane permeabilization and release of lysosomal proteins into the cytosol. Both, the subcellular distribution of enzyme activities and severity of pancreatitis were assessed in isolated acinar cells and mice following supramaximal secretagogue stimulation.

## Methods

### Materials

Collagenase from the bacteria *Clostridium histolyticum* was purchased from Serva (14,007). Enterokinase from the porcine intestine (E0885), Cholecystokinin (CCK) (C-9901), propidium iodide (p4170), and caerulein (C9026) were from Sigma Aldrich. Gly-Phe-β-napthylamide (GPN) was from Santa Cruz (sc-252858) and l-leucyl-l-leucine methyl ester was from Sigma Aldrich (BCCD0329). For enzyme kinetic measurements, the substrates for cathepsin B (AMC-Arg_2_, 4,004,789), cathepsin L (AMC-Phe-Arg, 4,003,379), and cathepsin C (H-Gly-Arg-AMC, 4,002,196) were purchased from Bachem. For trypsin activity measurement, the substrates were obtained from BioTrend (R110-Ile-Pro-Arg, 10,208) and Enzo Life Science (Boc-Gln-Ala-Arg-AMC, BML-P237-0005). Ketamine and Xylazine for mice anaesthetization were purchased from Selectavet. The lipase (LIPC, 03029590322) and amylase (Amyl Gen.2, 03183742122) measurement kits were obtained from Roche.

### Antibodies

The following antibodies were used for immunoblot analysis and immunofluorescence labeling: anti-cathepsin C (dilution: 1:1000, sc-5647, Santa Cruz Biotechnology, RRID: AB_2086961), anti-trypsin (dilution: 1:1000, sc-137077, Santa Cruz Biotechnology, RRID: AB_2300318), anti-amylase (dilution: 1:1000, sc-46657, Santa Cruz Biotechnology, RRID: AB_626668), anti-cathepsin B (dilution: 1:1000, MAB965, R&D systems, RRID: AB_2086935), anti-cathepsin L (dilution: 1:1000, MAB9521, R&D systems, RRID: AB_2087829), anti-α/β-tubulin (dilution: 1:1000, #2148, Cell Signaling, RRID: AB_2288042), anti-Rab7 (dilution: 1:1000 (for Immunoblot) and 1:100 (for immunofluorescence), #9367, Cell Signaling, RRID: AB_1904103), anti-syncollin (dilution: 1:1000, ab178415, Abcam), anti-LAMPI (dilution: 1:1000, ab24170, Abcam, RRID: AB_775978), anti-GAPDH (dilution: 1:1000, H86504M, Meridian Life Science, RRID: AB_151542), anti-LIMPII (dilution: 1:1000, NB400-129, Novus Biologicals, RRID: AB_2301298), anti-LAMPII (dilution: 1:1000, L0668, Sigma Aldrich, RRID: AB_477154), anti-rat (dilution: 1:16,000, HAF005, R&D systems, RRID: AB_1512258), anti-goat (dilution: 1:16,000, sc-2020, Santa Cruz Biotechnology, RRID: AB_631728), anti-rabbit (dilution: 1:16,000 (for immunoblots) or 1:200 (for immunofluorescence), NA934V, Amersham) and anti-mouse (dilution: 1:16,000, NA931V, Amersham).

### Induction of acute pancreatitis in vivo

All animal experiments were performed according to the national guidelines and animal facility protocols after prior approval by the institutional animal care facility (Landesamt für Landwirtschaft, Lebensmittelsicherheit und Fischerei (LALLF), Mecklenburg-Vorpommern). Wildtype C57BL/6J mice (RRID: IMSR_JAX:000664) were obtained from Charles River Laboratories (Sulzfeld, Germany) and B6.129(Cg)-Rab7^tm1.1Ale^/J mice (RRID: IMSR_JAX:021589) by The Jackson Laboratory (Bar Harbor, USA). CTSC^−/−^ mice were kindly provided by Dr. Christine Pham (Washington University School of Medicine, St. Louis, USA) [18], and Ptf1a^tm1.1(cre)Cvw^/J mice (RRID: MMRRC_000435-UNC) by Dr. Roland Schmid (Technische Universität München, Germany) [19]. B6.129(Cg)-Rab7^tm1.1Ale^/ Ptf1a^tm1.1(cre)Cvw^/J mice were obtained by mating B6.129(Cg)-Rab7^tm1.1Ale^/J mice with Ptf1a^tm1.1(cre)Cvw^/J mice. CTSB^−/−^ mice with a C57BL/6 background were maintained in our animal facility [20]. Acute pancreatitis was induced in 8–12 weeks-old mice by hourly intraperitoneal injections of 50 µg/kg body weight caerulein given once (1 h), four times (4 h) or eight times (8 h). Mice were sacrificed one hour after the last caerulein injection. The organs and blood were asservated immediately. One part of each sampled organ was frozen in liquid nitrogen and stored at −80 °C, one part was fixed in 4.5% formalin for paraffin embedding, and the last part was embedded in TissueTec O.C.T. compound (Sakura Finetek, Alphen aan den Rijn, The Netherlands). Serum samples were collected and stored at −80 °C. For GPN application, the mice were intravenously injected with 200 µg/kg body weight GPN (0.1% in DMSO) in 0.9% sodium chloride 1 h before and simultaneously with the first caerulein injection. Control mice received the vector solution without GPN.

Pancreas homogenates were prepared by using a Dounce homogenizer. Cell suspensions were then sonicated two times for 10 s, centrifuged at 20,800 g at 4 °C for 10 min, and used for further analysis.

### Acinar cell isolation and stimulation

Acinar cell preparation was performed by collagenase digestion as described previously [21]. The cells were cultured in Dulbecco’s modified Eagle medium containing 10 mM HEPES and 2% BSA. For stimulation of the cells, 1 µM CCK was added to the medium. Cellular necrosis and protease activity were measured for 20 min after CCK stimulation, while untreated cells served as controls. For in vivo protease measurement, living cells were maintained in a medium that contains 24.5 mM Hepes (pH 7.5), 96 mM sodium chloride, 6 mM potassium chloride, 1 mM magnesium chloride, 2.5 mM sodium dihydrogen phosphate, 500 µM calcium chloride, 11.5 mM glucose, 5 mM sodium pyruvate, 5 mM sodium glutamate, 5 mM sodium fumarate, 1% BSA, and 1% ethanol. Protease activity was measured using 10 µM R110-isoleucine-proline-alanine for trypsin, 20 µM AMC-phenylalanine-arginine for CTSL, or 20 µM AMC-arginine-arginine for CTSB. Necrosis was quantified by measuring the fluorescent intensity of propidium iodide intercalation into free DNA. For this purpose, 200 µl of cell medium containing a final concentration of 7.5 µM propidium iodide was added to acinar cells. After a resting period of 10 min cells were dissolved by repeated pipetting of the cell suspension and measured in a 96-well plate leader with a wavelength of λ_ex_ = 340 nm and λ_em_ = 620 nm. For GPN and LLOMe treatment of isolated acinar cells, cells were pre-treated with the substrate at the desired concentration for 30 min before CCK stimulation.

### Subcellular fractions

Subcellular fractionation was performed immediately after sacrification of mice using a modified protocol as described previously [22, 23]. After the removal, the pancreas was shredded in a homogenization buffer containing 240 mM sucrose, 5 mM MOPS, and 1 mM magnesium sulfate, set up to pH 6.5. Afterwards, the suspension was homogenized using a Dounce homogenizer wide A and B. The cell suspension was centrifuged at 150 g for 10 min at 4 °C to obtain the perinuclear fraction, 470 g for 15 min at 4 °C for the zymogen granule enriched fraction, 12,200 g for 12 min at 4 °C for the lysosome enriched fraction and 20,800 g for 10 min at 4 °C for the cytosolic fraction. After every step, the supernatant was used for the next centrifugation step. The pellet containing the aimed fraction was suspended in PBS, sonicated twice for 10 s, then snap frozen in liquid nitrogen and stored at −80 °C until further analysis.

### Biochemical assays

Serum amylase and lipase measurements were performed using the photometric Amyl2 and LipC kit from Roche as a kinetic over 30 min at 37 °C. Protease activity was measured in pancreas homogenates or subcellular fractions of the pancreas using fluorometric substrates as kinetics at 37 °C over 1 h. CTSB and CTSL were measured using a buffer containing 100 mM sodium acetate, 5 mM calcium chloride, and 10 mM DTT with a pH of 5.5 or 4, respectively, after adding 10 mM of AMC-Arg_2_ for CTSB or AMC-Phe-Arg for CTSL. CTSC kinetic was performed using a buffer containing 50 mM tri-sodium citrate-dihydrate at pH 6, 2 mM DTT, and 10 mM H-Gly-Arg-AMC. Trypsin activity was measured using a 100 mM Tris and 5 mM calcium chloride buffer at pH 8 and the substrates R110-Ile-Pro-Arg or Boc-Gln-Ala-Arg-AMC with a concentration of 10 mM. Determination of total trypsin content was performed by pre-activation of trypsinogen by enterokinase (10 milliunits) at 37 °C for 30 min. Protein content determination was performed using a Bradford assay to correct protease activation. 

### Western blot

Samples for western blot analysis were resuspended in PBS and sonicated twice for 10 s. Protein concentrations were determined by Bradford assay. SDS-PAGE and Western Blots were performed as described before [24]. Each sample was used in an equal amount. Blotting was performed using a nitrocellulose membrane and a transfer buffer according to Towbin (25 mM Tris, 192 mM glycine, 1% SDS, 10% methanol, pH 8.3) [25]. The membrane was blocked in NET buffer (0.15 M sodium chloride, 5 mM EDTA, 50 mM Tris–HCL, 0.05% Triton-X100) containing 0.2% gelatine. Primary antibodies were diluted 1:1000 and secondary antibodies 1:16,000 in the blocking buffer. Membranes were analyzed using the Super Signal West Femto Chemiluminescence kit or Super Signal Pico Chemiluminescence kit (Thermo Scientific, Waltham, USA). Membranes were additionally stained using Amido black (Merck, Darmstadt, Germany). Densitometric quantification of bands corresponding to active CTSB, single and heavy chain CTSL, and light chain CTSL was performed using Fiji [26].

### Histology

Paraffin-embedded slides were used for histological analysis by hematoxylin and eosin staining. Tissue damage was analyzed and scored using a modified score adapted from Niederau et al. [27], which was based on a quantification of necrosis, immunological infiltration, and edema. For immunohistochemical staining of Rab7 in pancreatic tissue, antigen retrieval was performed using the antigen retrieval solution of DAKO (Carpinteria, USA). PBS with 20% fetal bovine serum and 0.01% Triton X-100 was used for blocking. The primary antibody was diluted 1:100 and the secondary antibody 1:200 in the blocking buffer. The DAB substrate kit from Vector Laboratories (SK-4100) was used for chemical analysis.

### Statistical analysis

Statistical analysis was performed using GraphPad Prism (GraphPad Software, Boston, USA, RRID: SCR_002798). All data are presented with ± standard error. Every dataset was tested for outliers with the ROUT method and for normal distribution with the Kolmogorov–Smirnov test. For normally distributed data, the unpaired two-tailed Student’s *t-test* and for not normally distributed data or data where the statistical population is too small, the Mann–Whitney test was used for statistical analysis. For comparison of more than two groups, a Kruskal–Wallis test and Dunn’s multiple comparisons test was performed. Differences were considered statistically significant at a level of p < 0.05.

## Results

### Lysosomal protease activation and cellular damage in Rab7 deficient acinar cells upon supramaximal CCK stimulation

**Fig. 1:**
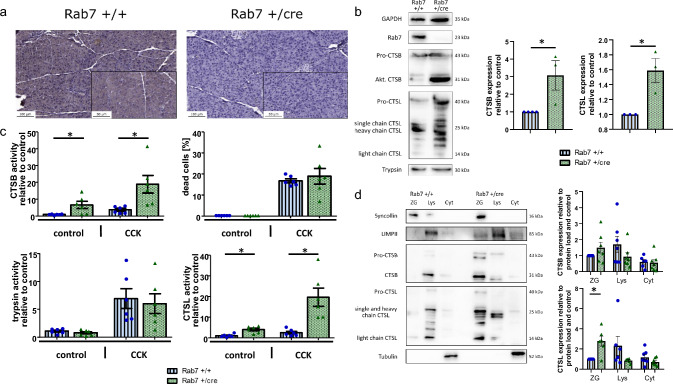
Rab7 and cathepsin B and L expression in pancreatic tissue and enzyme kinetics in isolated acinar cells stimulated with supramaximal concentrations of CCK. **a **Immunohistochemical staining in paraffin-embedded slides of pancreatic tissues with intraacinar Rab7 expression in control mice (Rab7 + / +) but no expression in the knockout mice (Rab7 + /cre). **b** Immunoblot analysis of pancreas homogenates shows an increased expression of cathepsin B and L in the Rab7 knockout mice. **c** Enzyme kinetics in living isolated acinar cells in response to supramaximal CCK in Rab7-KO (Rab7 cre/ +) and control mice (Rab7 fl/fl). CTSB and CTSL activities are significantly increased in Rab7 KO acinar cells, while trypsin and cell death remain unchanged. **d** Cathepsin B and L expression in subcellular fractions of the pancreas depends on Rab7. Both pro- and mature CTSB and CTSL are elevated in the zymogen-enriched “heavy fraction” of the Rab7 knockout pancreas. At least three animals were used for these experiments and the measurements were performed in triplicates. Values are mean ± SEM. * denotes p < 0.05

The absence of Rab7 in pancreatic acinar cells of Rab7 + /cre mice was confirmed by immunohistochemistry in paraffin-embedded pancreas slides using a Rab7-specific antibody. In contrast, there was a diffuse Rab7 expression inside acini of B6.129(Cg)-Rab7^tm1.1Ale^/J mice (in the following denoted as Rab7+/+ mice) (Fig. 1a). Immunoblot analysis from pancreas homogenates of Rab7 + / + and B6.129(Cg)-Rab7^tm1.1Ale^/ Ptf1a^tm1.1(cre)Cvw^/J mice (in the following denoted as Rab7 + /cre mice) showed only a weak Rab7 signal in the knockout mice, which is explained by the preserved Rab7 expression in ductal or endocrine cells of the pancreas while Rab7 deletion in acini was implemented by the p48 promotor (Fig. 1b).

Both, cathepsin B (CTSB) and L (CTSL) are central regulators of protease activation in acute pancreatitis. CTSB proteolytically activates trypsinogen [8], counteracted by CTSL [28]. Therefore, we investigated whether the absence of Rab7 in acinar cells modifies their protein expression and enzymatic activity. In immunoblot analysis of pancreatic tissue homogenates using an antibody against inactive pro-enzyme as well as mature CTSB, the signal intensity of a band around 31 kDa, corresponding to mature CTSB, was strongly enhanced while pro-CTSB expression remained similar (Fig. 1b). Signal intensities of mature CTSL, indicated by bands at 25 kDa and 14 kDa were also increased but in contrast to pro-CTSB, pro-CTSL expression was elevated, too. Likewise, total CTSB and CTSL enzyme activities in Rab7-depleted acinar cells were significantly higher than in controls. Upon supramaximal CCK stimulation for 20 min, intracellular CTSB and CTSL activities strongly increased in Rab7 + /cre acini while this increment was less pronounced in controls (Fig. 1c). Intracellular trypsinogen activation and pancreatic injury measured by cellular necrosis remained comparable for both groups indicating that induction of acute pancreatitis still occurs despite loss of Rab7. Based on the observation of elevated lysosomal hydrolase activities in Rab7cre/ + mice we were interested in their subcellular location. In subcellular fractions of pancreas homogenates generated by gradient density centrifugation, we determined protein expression and enzymatic activity of CTSB and CTSL. While mature CTSB was mainly expressed in lysosomes and only weakly in the zymogen-enriched fraction of control pancreas, there was a much stronger CTSB signal in the secretory compartment of Rab7cre/ + mice. Similarly, higher expression of CTSL heavy and light chains were found in the heavy fraction. Syncollin, LIMPII, and tubulin served as markers for the zymogen-enriched fraction, the lysosomal-enriched fraction, and the cytosolic fraction, respectively (Fig. 1d).

### Initiation of acute pancreatitis is independent of acinar Rab7 expression

**Fig. 2:**
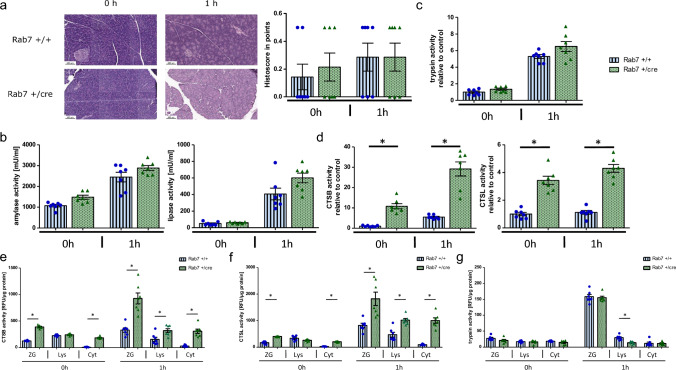
Initiation and early phase of acute pancreatitis in Rab7 knockout mice and controls. **a **HE stainings and histoscore indicate no difference in local damage in Rab7-KO mice. **b**, **c **Likewise, trypsin, serum amylase and lipase activities are not different at 0 h and 1 h. **d **In contrast, CTSB and CTSL activities are significantly increased at every time point, showing the same trend compared to control mice. **e** Subcellular fractionations of pancreatic tissue 1 h after caerulein injection display a significant increase of CTSB activity in the zymogen-enriched, lysosomal, and cytosolic fraction of Rab7 + /cre mice, whereas the lysosomal CTSB activity decreases in controls. **f** CTSL activity increases as well in all fractions after caerulein induction. **g** Trypsin activity at 0 h is at basal levels but increases in the zymogen-enriched fraction in both mouse strains upon caerulein. Cathepsin and trypsin activities are presented as relative fluorescence units (RFU) and are normalized for protein amounts. At least seven animals were used for these experiments and the measurements were performed in duplicates. Values are mean ± SEM. * denotes p < 0.05

To investigate the significance of Rab7 in acinar cells for onset of acute pancreatitis we induced acute pancreatitis using the caerulein model and compared protease activation and disease severity at 0 h and 1 h after disease onset. The severity of pancreatitis was identical in both Rab7 + /cre mice and corresponding controls as shown by total histological damage (Fig. 2a) and the activation of serum amylase and lipase (Fig. 2b). In line with our observations in the ex vivo experiments, activation of trypsin was also independent of Rab7 in the caerulein model (Fig. 2c). Moreover, the activities of the lysosomal proteases CTSB and CTSL, reflecting amounts of these proteins, were strongly increased in pancreatic homogenates of Rab7 + /cre mice during unstimulated conditions (0 h) and the early disease phase (Fig. 2d) similar to isolated and CCK-stimulated acinar cells. CTSB activities increased in both the zymogen and lysosomal fractions of Rab7 deficient mice while a re-distribution of CTSB activity as seen in controls did not occur (Fig. 2e). In addition, we detected a higher CTSB activity in the cytosolic pancreatic fractions of Rab7 depleted mice that further increased upon caerulein application. A similar trend was seen for CTSL, a trypsinogen inactivating enzyme (Fig. 2f). In contrast, intracellular trypsinogen activation remained constant irrespective of Rab7 expression and activities were almost exclusively detected in the secretory vesicle compartment (Fig. 2g). This activation even occurred despite the absence of CTSB-redistribution into the secretory compartment in Rab7 depleted mice.

### Deficiency of Rab7 increases the severity of acute pancreatitis at later stages

**Fig. 3:**
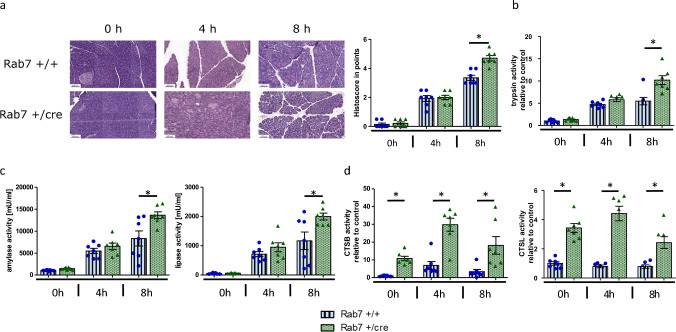
Severity of acute pancreatitis is increased at 8 h in Rab7 knockout mice. **a **HE-stained pancreas and histoscore analysis show an increased severity at 8 h. **b** Trypsin activity in pancreas homogenates is elevated at 8 h of AP. **c **Likewise, serum lipase and amylase activities are increased. **d **Total activities of CTSB and CTSL are elevated in pancreatic homogenates of Rab7 + /cre mice throughout unstimulated and stimulated conditions. At least seven animals were used for each experiment and all experiments were performed in duplicates. Values are means ± SEM. * denotes p < 0.05

Our results suggest that acute pancreatitis and organ injury begin despite perturbation of lysosomal function either by lysosomotropic substances or by inhibition of Rab7. During later disease stages both histopathological damage (Fig. 3a) and serum amylase, as well as lipase activities (Fig. 3c), were significant increased at 8 h of pancreatitis in Rab7 + /cre mice. Correspondingly, trypsinogen activation in pancreatic homogenates had the same trend (Fig. 3b). However, activities of CTSB and its counteracting enzyme CTSL, remained constantly elevated during all time points including the unstimulated condition (0 h) when compared to controls (Fig. 3d).

### Disruption of the lysosomal membrane does not alter the onset of the disease in vitro or in vivo

**Fig. 4:**
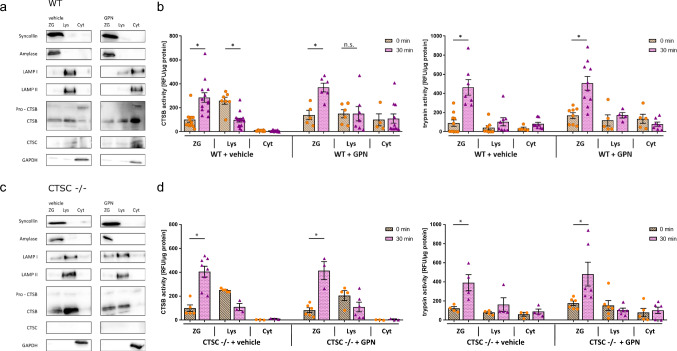
GPN disrupts lysosomal membranes but does not influence trypsin and CTSB activation after induction of secretagogue-induced pancreatitis. **a **Immunoblots of pancreatic subcellular fractions of WT mice (C57BL/6) show a redistribution of lysosomal proteins LAMP1, LAMP2, CTSB, CTSC from the lysosomal enriched fraction into the cytosolic fraction after GPN injection. **b **Lysosomal disruption does not prevent activation of trypsin and CTSB localized in the zymogen fraction (ZG) after caerulein injection. In contrast to untreated controls, there is no redistribution of CTSB activity from the lysosomal to the zymogen granule compartment under GPN. **c **No redistribution of lysosomal proteins is seen in CTSC^−/−^ mice. **d **Absence of CTSC prevents the lysosomotropic function of GPN and retains subcellular CTSB distribution and intracellular trypsin activation in CTSC^−/−^ mice. At least three animals were used for these experiments and the measurements were performed in triplicates. Values are mean ± SEM. * denotes p < 0.05

Our results demonstrate that the impairment of intracellular vesicle transport based on a loss of Rab7 does not prevent intracellular zymogen activation. In a next step, we sought to investigate whether a rupture of the lysosomal membrane by the lysosomotropic substrate GPN affects digestive protease activation and initiation of acute pancreatitis. GPN is hydrolyzed by the lysosomal enzyme CTSC leading to an osmotic lysis of the lysosomes [16]. Therefore, we injected mice twice with GPN, and one hour later we prepared subcellular fractionations of pancreas homogenates by gradient density centrifugation. As GPN is a substrate of CTSC and only the hydrolysis products cause the lysosomal disruption, we used CTSC knockout mice as controls when investigating GPN-dependent protease activation and pancreatic injury. Subcellular fractionation followed by immunoblot analysis in C57BL/6 wildtype mice showed a redistribution of the lysosomal proteins LAMP I, LAMP II, CTSB, and CTSC from the lysosomal enriched fraction into the cytosolic fraction denoting a disruption of the lysosomal membrane followed by a release of lysosomal contents into the cytosol. Syncollin and amylase, two zymogen marker proteins, stayed in the “heavy” secretory compartment that is not affected by GPN (Fig. 4a).

We next investigated subcellular localization of CTSB and trypsin depending on GPN in vivo. In C57BL/6 wildtype mice without GPN treatment, CTSB enzyme activity shifted from the lysosomal to the secretory compartment after 30 min of caerulein injection, while cytosolic CTSB activity remained at a very low level. In parallel, trypsin activity increased in the zymogen compartment (Fig. 4b). Additional GPN treatment still enabled an activation of CTSB localized in the secretory vesicles while CTSB activity in the lysosomal enriched fraction did not substantially change. Similar to controls, there was a concomitant trypsinogen activation in the zymogen granules of GPN treated mice. As a result of lysosomal membrane disruption by GPN CTSB activity was also detectable in the cytosol. Obviously, the elevation of CTSB activity cannot result from a shift of lysosomal CTSB activity to the secretory compartment and is rather the consequence of an activation of CTSB already localized inside the secretory compartment (Fig. 4b). As a control, we used CTSC^−/−^ mice that underwent GPN pre-treatment followed by caerulein-stimulation and preparation of subcellular fractions. CTSB and marker proteins showed the same subcellular distribution as vehicle treated mice (Fig. 4c). Upon caerulein, CTSB activity decreased in the lysosomal fraction along with an increase of CTSB in the secretory compartment and was completely independent of GPN (Fig. 4d). In parallel, trypsinogen activation remained unaffected as well in CTSC^−/−^ mice after caerulein induction.

**Fig. 5:**
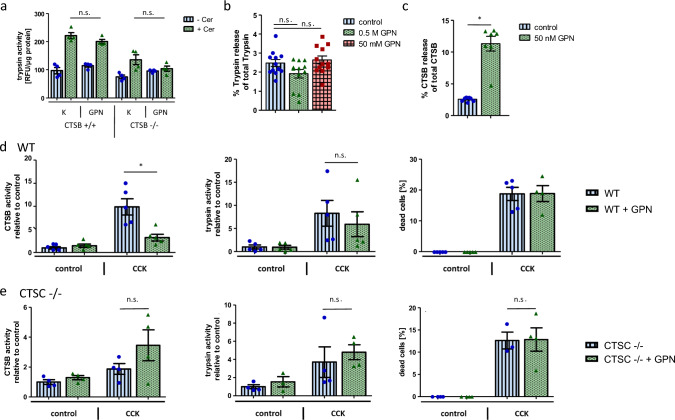
Trypsin and cathepsin B activation at onset of caerulein induced acute pancreatitis. **a **Trypsin activity in pancreatic homogenates increase in both GPN pre-treated and control mice upon caerulein but remain inactivated in CTSB^−/−^ mice, indicating that trypsinogen activation is dependent on presence of CTSB but not of GPN. **b **GPN does not permeabilize the membranes of zymogen-enriched fractions from WT pancreas, as even high GPN concentrations do not cause a release of trypsin into the supernatant. A Kruskal–Wallis test and Dunn’s multiple comparisons test was performed for statistical analysis. **c **In contrast, incubation of lysosome-enriched fractions with 50 nM GPN for 1 h induces a CTSB release from the lysosomes into the extracellular space. Enzyme activities are presented as relative fluorescence units (RFU). **d **In isolated and GPN pre-incubated wildtype (WT) acinar cells intracellular CTSB activity significantly decreases after 20 min of CCK stimulation but trypsin activity and cell death are unaltered. **e **CTSB activity does not significantly change after supramaximal CCK stimulation in isolated CTSC^−/−^ acinar cells following GPN. Trypsin activity and necrosis are also not unaffected. At least three animals were used for each experiment and all experiments were performed in duplicates. Values are means ± SEM. *denotes p < 0.05

To clarify the significance of CTSB for trypsinogen activation we repeated experiments in CTSB^−/−^ mice. In pancreatic homogenates of CTSB^−/−^ mice treated with GPN and caerulein, no trypsinogen activation occurred, strengthening the role of CTSB as a crucial trypsinogen-activator in secretagogue-induced pancreatitis (Fig. 5a). Due to the absence of CTSC in the secretory vesicles GPN cannot be hydrolyzed and their membranes are resistant to GPN even in high doses (50 mM) as trypsin release was not detectable in the supernatants (Fig. 5b). As expected, there is a strong CTSB release after incubation of the CTSC-containing lysosome-enriched subcellular fraction with GPN (Fig. 5c). Exposure of GPN to isolated living acinar cells led to a reduced intracellular CTSB activation upon supramaximal CCK stimulation but trypsinogen activation and cellular necrosis remained unchanged (Fig. 5d). When we repeated the experiments in CTSC deficient acinar cells the kinetics of CTSB and trypsinogen activation after CCK stimulation were not affected of GPN (Fig. 5e). These results indicate that zymogen activation inside the secretory compartment appears to be independent of the lysosome and lysosomal contents. Additionally, they support the critical function of cathepsin C as a prerequisite for GPN hydrolysis.

l-Leucyl-l-Leucine methyl ester (LLOMe) is another compound with lysosomotropic capabilities. Pre-incubation of isolated acinar cells with LLOMe confirmed our results with GPN as trypsinogen activation and necrosis have taken place despite a decrease of total intracellular CTSB activity upon CCK stimulation (Supplementary Fig. 1a). Again, neither CTSB nor trypsin activation and cellular necrosis were distorted in CTSC^−/−^ acinar cells, as presence of CTSC is also required for processing of LLOMe into its membranolytic form [29] (Supplementary Fig. 1b).

**Fig. 6:**
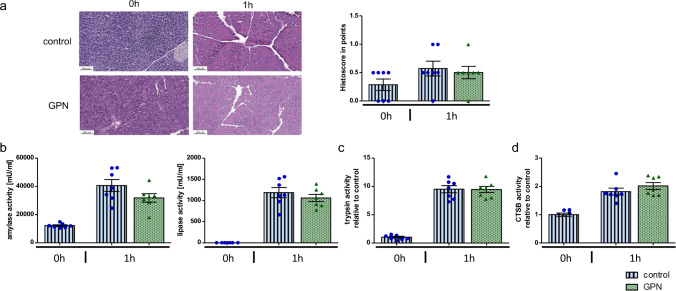
Initiation of acute pancreatitis is still maintained after disruption of lysosomes. **a **HE-stained pancreas and histoscore analysis demonstrate comparable severity at 1 h. **b **Serum lipase and amylase activities show no differences after 1 h. **c**, **d **Trypsin and CTSB activities in pancreas homogenates of GPN-treated mice are equal to controls. At least seven animals were used for each experiment and all experiments were performed in duplicates. Values are means ± SEM. * denotes p < 0.05

To elucidate the role of the lysosomotropic agent GPN and lysosomal disruption in early acute pancreatitis in vivo we induced acute pancreatitis by caerulein in C57BL/6 wildtype mice. One group of animals received an additional GPN treatment. The pancreas of the GPN-treated mice did not show any morphological abnormalities. Local pancreatic injury represented by histological damage in H.E. stained pancreata (Fig. 6a), serum amylase, and lipase levels (Fig. 5c) did not show differences in early pancreatitis (1 h). Accordingly, intrapancreatic trypsinogen activation was activated identically in GPN-treated mice and controls (Fig. 6b) and total CTSB activities in homogenates showed similar trends despite lysosomal permeabilization by GPN (Fig. 6d).

## Discussion

Acute pancreatitis is believed to be caused by premature activation of trypsinogen inside acinar cells [30]. Evidence for its crucial role is derived from experimental models in rodents as well as from clinical observations in humans and genetic studies [31–34]. Several investigations support the cathepsin B-dependent proteolytic activation of trypsinogen in experimental pancreatitis since depletion of CTSB in a knockout model or by pharmacological treatment markedly abolished trypsinogen activation and pancreatic damage [2, 8, 20]. As a prerequisite, both enzymes need to be co-localized in the same subcellular compartment but the underlying mechanisms are incompletely understood and are still a matter of debate. The mechanisms of co-localization are based on different hypotheses namely the fusion of the lysosomal and secretory compartment [9, 35], a missorting of lysosomal hydrolases from the Golgi [23, 36], trypsinogen activation in autophagolysosomes [14] or an event completely independent of autophagosome formation [37].

In the current study, we provide evidence by two experimental approaches that early intracellular trypsinogen activation occurs independently of the lysosomal compartment and exclusively takes place in the secretory vesicles. Despite a distortion of lysosomal traffic by Rab7 depletion and pharmacological perturbation of lysosomal integrity, the initiation of pancreatitis represented by trypsinogen activation and pancreatic injury was maintained, hence contradicting the concept of fusion of lysosomes with mature zymogen granules as a *conditio sine qua non* for the onset of acute pancreatitis. CTSB stored in the secretory compartment appears to be sufficient for this primary activation step in acute pancreatitis.

Our results are consistent with observations from a previous study that showed an abundant CTSB activity in secretory vesicles even under physiological conditions, even prior to the onset of acute pancreatitis. Following a supramaximal caerulein stimulus, CTSB activity increases along with regular processing of CTSB precursors to their mature form inside the secretory vesicle-containing fraction supporting the concept that not only a shift of CTSB activity or processed protein from the lysosomal fraction but also a true activation of CTSB takes place in that compartment [20].

Although the premature and intracellular trypsinogen activation apparently occurs independently of lysosomal CTSB the presence of this cysteine protease is still essential. Trypsin activation is completely abolished when CTSB is deleted as demonstrated in the CTSB^−/−^ mice. In addition, an autoactivation of trypsinogen as a side effect due to lysosomotropic drugs could be ruled out. Moreover, due to the absence of CTSC in secretory vesicles, GPN did not harm their integrity as demonstrated in isolated zymogen granules.

The function of lysosomes for acute pancreatitis needs to be re-evaluated. Inefficient lysosomal function with an absent or retarded fusion with zymogens and the more severe course at later disease stages in Rab7 mice suggest that the intracellular fusion of lysosomes with vesicular organelles rather helps to mitigate pancreatitis and to restore from pancreatic injury by removing damaged organelles. Defects in intracellular trafficking were suspected to be causative for other inflammatory disorders such as rheumatoid arthritis [38, 39] and gastritis [40]. Furthermore, mice with deficiency in lysosome-associated membrane protein 2 (LAMP-2) spontaneously developed increased levels of mature trypsin and histopathologic injury characteristic of acute pancreatitis accompanied by impaired autophagy of the exocrine pancreas [41]. In view of their critical function for maintenance of structural and functional integrity and regulation of fusion with other organelles [42] deficiency of LAMP-2 will ultimately lead to lysosomal dysfunction. Moreover, loss of LAMP-2 does not preclude initiation of pancreatitis as LAMP-2 null mice developed acute pancreatitis upon caerulein stimulation questioning again the role of lysosomes for initiation of acute pancreatitis.

The simultaneous increase of CTSB and CTSL activities in Rab7 deficient pancreas might be a reason why trypsin activity eventually did not differ under unstimulated conditions because the balance of these two counteracting proteases is still maintained. CTSL is a known trypsinogen and trypsin degrading enzyme thus antagonizing the function of CTSB [28]. The increases in activity seem to result from an endogenously increased protein expression in Rab7-deficient pancreas that was shown in a previous study using Rab7-deficient pancreas homogenates [43]. In pancreas-specific CTSB and CTSL double knockout undergoing with caerulein-induced acute pancreatitis intrapancreatic trypsin activity was increased while disease severity was not affected when compared to wildtypes or mice with pancreas-specific single CTSB or CTSL knockout [4]. These findings indicate that CTSL-mediated degradation exceeds trypsinogen and trypsin activation by CTSB. At first appearance, these results seem to be at odds with our observations of increased trypsin activity despite increased CTSB and CTSL activities. However, we found a much higher elevation of CTSB than of CTSL after 8 h of caerulein injections in pancreas-specific Rab7-deficient mice. It can be speculated that the activating effects of CTSB outweighed the degrading effects of CTSL. Moreover, we cannot rule out an additional trypsinogen activation caused by invading macrophages phagocytosing acinar cell components [44].

Our experiments cannot not entirely clarify the nature of the subcellular compartment where protease activation begins. Subcellular separation by sucrose gradient density centrifugation leads to a comparatively heavy product that eventually contains other subcellular components besides zymogens and is therefore termed as zymogen-enriched fraction. Cytoplasmic vacuoles have been recognized as an early sign of acute pancreatitis and there is much evidence, that they represent autophagic vacuoles containing partially degraded material and digestive enzymes, in which trypsinogen activation occurs [14, 45, 46]. However, autophagic vacuoles are not observed until later stages of the disease as reported by several groups, and thus do not seem to be the underlying reason for the intracellular distribution of cathepsins in Rab7-deficient mice. Other concepts mention an initial activation of trypsin in secretagogue-induced pancreatitis in a subcellular compartment of the secretory compartment neither involving autophagosomes, endosomes, nor lysosomes [37].

The question that remains is, how CTSB stored in secretory granules is endogenously inhibited before it causes trypsinogen activation. The optimal catalytic activity of CTSB is linked to acidic pH conditions and therefore the maintenance of specific pH conditions inside the vesicular compartment can prevent premature CTSB processing. Ex vivo experiments using the V-type ATPase inhibitor bafilomycin A1 as a pH-neutralizing agent clearly demonstrated that prevention of acidification also precluded from CTSB processing to the mature enzyme [20]. Secondly, cysteine protease inhibitors, localized inside of zymogen granules, inhibit premature CTSB activation. Cystatin C is located not only in the cytosol but also in a small amount in the secretory compartment [20]. Their presence inside vesicular compartments might be an additional explanation for the absent trypsinogen activation in unstimulated pancreas despite higher endogenous CTSB and CTSL activities in unstimulated Rab7 mice. Furthermore, an extra-acinar trypsinogen activation emerges at later disease stages as infiltrating macrophages can activate trypsinogen due to their own CTSB expression which contributes to total intrapancreatic trypsin activity and organ damage [44]. The role of CTSB localized into the cytosol is still under debate. Previous studies mention pro-apoptotic effects of cytosolic CTSB in acinar [7] and in other cell types [47]. Others have argued that vesicular but not cytosolic CTSB can induce apoptosis by activation of trypsin since CTSB activity is quenched by cystatin B and C in the cytosol [20]. Whether CTSB leaked into the cytosol eventually contributes to the less favorable course of acute pancreatitis in Rab7 mice in the later disease course needs to be clarified further.

There are limitations of our study. First, functions of the Rab7 protein are much more complex since this small GTPase is not only expressed on lysosomes but also on late endosomes targeted to lysosomes [48]. For that reason, Rab7 is also important for the transport of cellular cargo into late endocytotic compartments such as late endosomes, lysosomes, and finally autophagosomes [13] and a functional impairment of other subcellular vesicles rather than zymogen granules cannot be completely excluded. Being a part of the so-called Rab5-to-Rab7 switch of endosomes during the maturation of early Rab5-positive endosomes to Rab7-positive late endosomes, Rab7 is important for endosomal and lysosomal biogenesis [49]. Furthermore, Rab7 is even involved in the retrograde transport of cargo from the endosomal network into the trans-Golgi network [50], in mitophagy [51] and in lipophagy [52]. The insertion of the loxP sites, flanking the exon 1 of the Rab7 gene neither results in an absence of Rab7 expression nor an abnormal phenotype of these mice [53]. Therefore, we have chosen Rab7 flox/flox mice as controls. However, it cannot entirely ruled out, that genetic modifications by loxP-flanked alleles do not influence physiology of pancreatic acinar cells and disease pathogenesis at all or exclude any excision activity beyond the intended tissue [54–56]. Our findings can neither prove nor refute whether a co-localization of the lysosomal and secretory compartment occurs at all, but they indicate that lysosomes seem to be dispensable for the early moments of acute pancreatitis. Experiments with prolonged incubation of lysosomotropic substances would be helpful to elucidate their role in later stages of acute pancreatitis in vivo. However, their toxicity limits in vivo experiments for longer time periods [57, 58]. At least our experiments demonstrated that even high doses of GPN do not harm the integrity of the secretory compartment when incubated for short-term. Furthermore, CTSC-independent effects of GPN need to be considered, as GPN can transiently increase the cytosolic pH linked with a Ca^2+^ release from the ER into the cytosol. These cellular responses might also influence disease pathogenesis in isolated acinar cells and in mice [59]. Finally, the role of CTSB-mediated trypsinogen activation as the disease-relevant mechanism for acute pancreatitis needs to be re-assessed. Investigations in mouse models carrying trypsinogen mutations with blocked trypsinogen autoactivation but preserved or even enhanced CTSB-driven activation showed no correlation between disease severity and intrapancreatic trypsin levels in caerulein pancreatitis. Thus changes in the amount of CTSB-mediated intrapancreatic trypsin eventually may have no effect on the disease response at least in the caerulein model for pancreatitis [3, 60].

In summary, we were able to provide evidence for trypsinogen activation independent of CTSB localized in lysosomes. This activation is particularly driven by CTSB stored endogenously in the secretory compartment. We therefore conclude that lysosomes and lysosomal-zymogen granule fusion, respectively, may play a minor role in the initiation of the disease.

### Supplementary Information

Below is the link to the electronic supplementary material.

Supplementary Figure 1: Effect of the lysosomotropic compound LLOMe on protease activation. a: Similar to GPN, CTSB activation is reduced but trypsinogen activation and cell death are preserved in LLOMe pre-treated and CCK stimulated isolated acinar cells. b: In CTSC-/- acinar cells, LLOMe has no effect on CTSB and trypsin activation as well as cell death. At least four animals were used for each experiment and all experiments were performed in triplicates. Values are means ± SEM. * denotes p<0.05. Supplementary file1 (PPTX 176 KB)

## Data Availability

The authors declare that all supporting data of this study are provided within the figures and the supplementary information. If any raw data or further information is needed, they are available from the corresponding author on reasonable request.
