# Supplementary figures and images for: Initiation of acute pancreatitis in mice is independent of fusion between lysosomes and zymogen granules
Source: Cell Mol Life Sci. 2024 May 6;81(1):207. doi: 10.1007/s00018-024-05247-7 (PMC11074023; doi:10.1007/s00018-024-05247-7)

## Slide 1
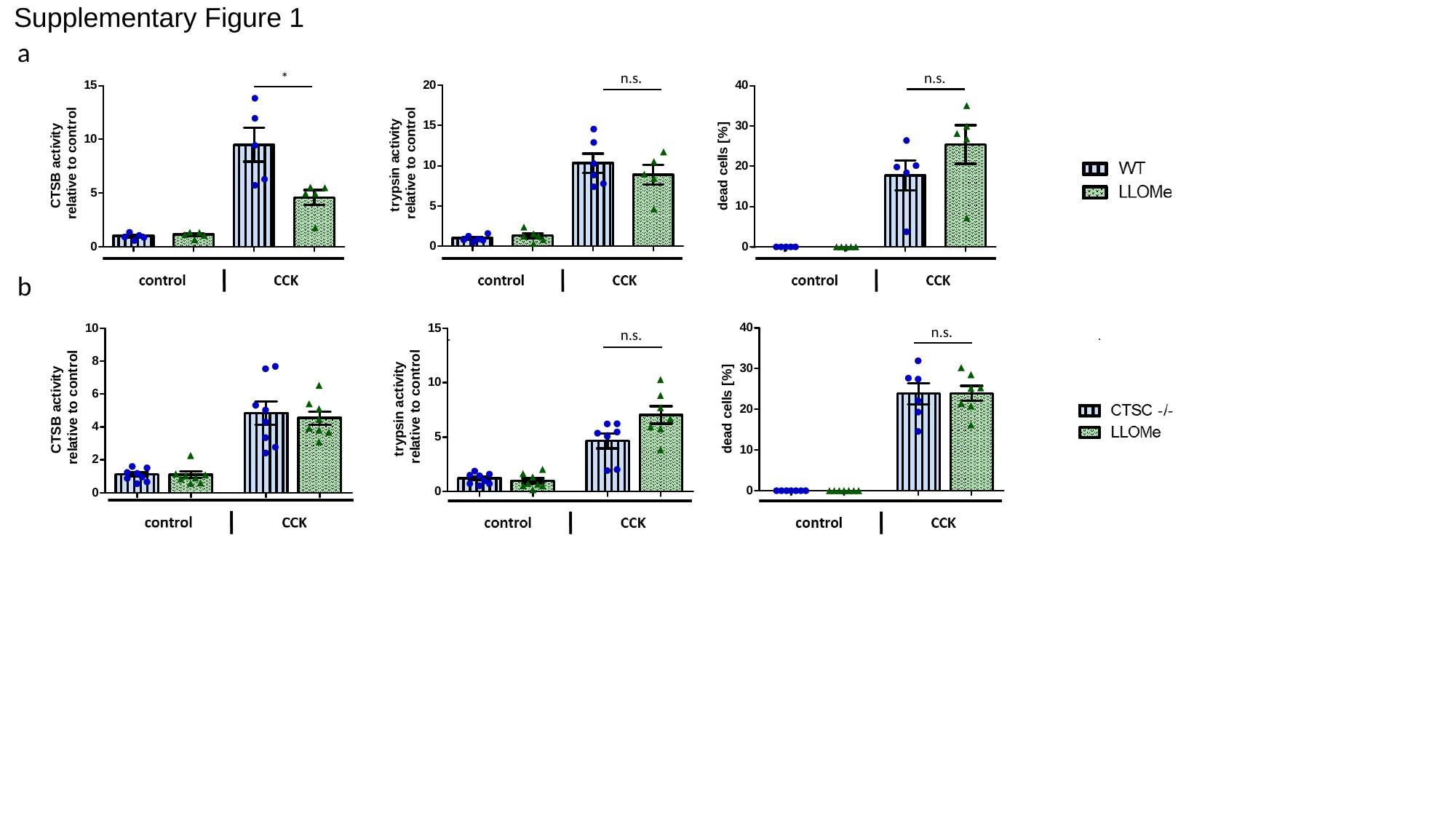

Supplementary Figure 1
a
n.s.
*
n.s.
b
n.s.
n.s.

Supplement: Supplementary file 1 — Supplementary Figure 1: Effect of the lysosomotropic compound LLOMe on protease activation. a: Similar to GPN, CTSB activation is reduced but trypsinogen activation and cell death are preserved in LLOMe pre-treated and CCK stimulated isolated acinar cells. b: In CTSC-/- acinar cells, LLOMe has no effect on CTSB and trypsin activation as well as cell death. At least four animals were used for each experiment and all experiments were performed in triplicates. Values are means ± SEM. * denotes p<0.05. Supplementary file1 (PPTX 176 KB) [file 18_2024_5247_MOESM1_ESM.pptx]
